# Polymeric Amines and Ampholytes Derived from Poly(acryloyl chloride): Synthesis, Influence on Silicic Acid Condensation and Interaction with Nucleic Acid

**DOI:** 10.3390/polym9110624

**Published:** 2017-11-16

**Authors:** Elena N. Danilovtseva, Uma Maheswari Krishnan, Viktor A. Pal’shin, Vadim V. Annenkov

**Affiliations:** 1 Limnological Institute of the Siberian Branch of the Russian Academy of Sciences, 3, Ulan-Batorskaya St., P.O. Box 278, 664033 Irkutsk, Russia; danilovtseva@yahoo.com (E.N.D.); acrom@mail.ru (V.A.P.); 2 Centre for Nanotechnology & Advanced Biomaterials (CeNTAB), School of Chemical and Biotechnology, SASTRA University, Thanjavur 613401, Tamil Nadu, India; umakrishnan@sastra.edu

**Keywords:** poly(acryloyl chloride), polyamines, acid-base properties, composites, silica, gene delivery

## Abstract

Polymeric amines are intensively studied due to various valuable properties. This study describes the synthesis of new polymeric amines and ampholytes by the reaction of poly(acryloyl chloride) with trimethylene-based polyamines containing one secondary and several (1–3) tertiary amine groups. The polymers contain polyamine side chains and carboxylic groups when the polyamine was in deficiency. These polymers differ in structure of side groups, but they are identical in polymerization degree and polydispersity, which facilitates the study of composition-properties relationships. The structure of the obtained polymers was confirmed with ^13^C nuclear magnetic resonance infrared spectroscopy, and acid-base properties were studied with potentiometry titration. Placement of the amine groups in the side chains influences their acid-base properties: protonation of the amine group exerts a larger impact on the amine in the same side chain than on the amines in the neighboring side chains. The obtained polymers are prone to aggregation in aqueous solutions tending to insolubility at definite pH values in the case of polyampholytes. Silicic acid condensation in the presence of new polymers results in soluble composite nanoparticles and composite materials which consist of ordered submicrometer particles according to dynamic light scattering and electron microscopy. Polymeric amines, ampholytes, and composite nanoparticles are capable of interacting with oligonucleotides, giving rise to complexes that hold promise for gene delivery applications.

## 1. Introduction

Polymeric amines have wide-ranging applications as emulsifiers, components of pharmaceutical preparations, in cation exchange resins, as matrices for composites and catalysts [1,2,3], and in drug and gene delivery systems [4,5,6,7,8,9]. Carbochain polymeric amines, e.g., poly(vinyl amine) are often synthesized by means of polymer-analogous reactions (modification of the existing polymer) [10]. This is due to the high activity of the amine groups that results in difficulties in monomer purification and polymerization. Moreover, some monomers (vinyl amine) do not exist in a stable form. When polymer-analogous reactions are carried out under moderate conditions without the destruction of the main polymeric chain, it is possible to obtain a set of (co)polymers with the same polymerization degree and various ratios of the functional groups. Such sets of (co)polymers are interesting in the context of investigating the composition–property relations as freezing one parameter (polymerization degree) allows one to concentrate solely on the composition.

**Figure 1:**
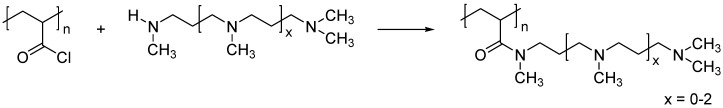
Scheme of the polymer synthesis.

This work aimed at the synthesis of polymeric amines and ampholytes starting from poly(acryloyl chloride) (PAC). This polymer can be readily obtained by radical polymerization of the monomer [11]. The chloro anhydride group readily reacts with primary and secondary amines, which allows the introduction of various substituents through the amide group. Unfortunately, this polymer is rarely applied in polymer-analogous reactions. We used polyamines containing one secondary and several tertiary amine groups in the reaction with PAC (Figure 1).

These amines were obtained by a stepwise procedure [12] which we designed with the objective to synthesize polyamines simulating substances found in diatom algae [13]. Variation of the amine:–COCl ratio allowed us to obtain completely substituted homopolymers and polyampholytes after the hydrolysis of the residual chloro anhydride groups. An alternative way to obtain these polymers is the post-polymerization modification of macromolecules from the activated esters of acrylic acid [14]. This method has some disadvantages when compared with the PAC approach, activated esters of acrylic acid are relatively expensive substances and the modification reaction requires an elevated temperature and activators [15], which could result in the destruction of the main polymeric chain.

The new polymers were studied as activators of silicic acid condensation, giving rise to soluble composite nanoparticles and solid materials. The interaction of the polymeric amines and composite nanoparticles with DNA oligonucleotide was also studied to demonstrate the potential of these compounds for gene delivery applications.

## 2. Materials and Methods 

### 2.1. Materials

2,2′-Azobis(2-methylpropionitrile) (AIBN) (Sigma-Aldrich, St. Louis, MO, USA) was recrystallized from ethanol prior to use. Dioxane (Sigma-Aldrich) was distilled under sodium. Dimethylformamide (DMFA) (Sigma-Aldrich) was dried with CuSO_4_ (30 min) and distilled at 5 mm Hg. Polyamines containing 2–4 nitrogen atoms (N2, N3, and N4 amines, respectively) were obtained according to procedures described in [12]. Acryloyl chloride (Sigma-Aldrich) was distilled before polymerization. Fluorescein 3′-tagged DNA oligonucleotide GATCTCATCAGGGTACTCCTT was purchased from Evrogen JSC (Moscow, Russia). Chemicals for agarose gel electrophoresis were obtained from DIAEM JSC (Moscow, Russia). Na_2_SiO_3_·5H_2_O, 1 and 0.1 M HCl solutions, trifluoroacetic acid (TFA), HEPES [(4-(2-hydroxyethyl)-1-piperazineethanesulfonic acid) and reagents for the molybdenum blue assay (ammonium molybdate, oxalic acid, 4-methylaminophenol sulfate, sodium sulfite, standard silicate solution, hydrochloric acid (35%), and sulfuric acid (98%)) were purchased from Sigma-Aldrich (St. Louis, MO, USA), Fisher(Hampton, NH, USA), or Acros (Geel, Belgium) chemicals and used without further treatment. The yeast *Saccharomyces cerevisiae*, parent-type strain W303-1B (*MATα ade2–1 his3–11, 15 trp1–1 leu2–3112 ura3–1 [rho+]*) was procured from the collection of the Siberian Institute of Plant Physiology and Biochemistry (SIPPB SB RAS, Irkutsk, Russia). 

### 2.2. Characterization of the Copolymers and Composites

Fourier transform infrared (FTIR) spectra were recorded with an Infralum FT-801 spectrometer (Simex JST, Novosibirsk, Russia) using an attenuated total reflection (ATR) attachment (for polymers) or KBr pellets (for composites). ^1^H and ^13^C Nuclear magnetic resonance (NMR) spectra were obtained on a Bruker DPX 400 spectrometer (400.13 and 100.61 MHz, respectively, Billerica, MA, USA) in D_2_О. Relaxation delays (D1) between pulses in ^13^C NMR were 10 s, which according to preliminary experiments, allowed us to prevent the influence of relaxation effects on the integral intensity of the ^13^C NMR peaks. This is more than five times greater than the relaxation times in acrylic polymers [16]. Potentiometry measurements were performed on a “Multitest” ionometer using a combined pH-electrode in a temperature-controlled cell at 20 ± 0.02 °C. Solutions of 1.5 g∙L^−1^ were prepared for potentiometry titration. A solution of 0.1 M HCl was used for adjusting the pH of the solutions up to 2.8, and 0.1 M NaOH was employed as a titrant.

The molecular mass of the new polymers was estimated via size-exclusion chromatography (SEC) using a Milichrom A02 chromatograph (JSC Econova, Novosibirsk, Russia) with 2 mm × 75 mm column filled with SRT SEC-100 5 μm phase (Sepax Technologies, Inc., Newark, NJ, USA), operated at 35 °C using 10:90 methanol:water solution of TFA, pH 2.5. The flow rate of the mobile phase was set at 0.03 mL∙min^−1^ (pressure 100 psi), whereas the injection volume for 1 g∙L^−1^ of the sample solution was 1 μL. Fractionated samples of poly(vinyl formamide) (PVFA) [17] were applied as standards (*M*_w_/*M*_n_ < 1.3).

Dynamic light scattering (DLS) experiments were performed using a LAD-079 instrument built at the Institute of Thermophysics (Novosibirsk, Russia). The solutions were purified from dust using syringe filter units (0.45 μm pore size, Sartorius 16555-Q Minisart, Sartorius AG, Chöttingen, Germany). The experiments were performed at 20 ± 0.02 °C. Measurements were carried out with a 650-nm solid-state laser at a 90° scattering angle. Correlation functions were analyzed with a polymodal model using a random-centroid optimization method [18]. Zeta-potential (ζ) of the polymer-oligonucleotide complexes was measured with a Zetasizer ZS90 (Malvern Instruments Ltd., Malvern, UK).

Transmission electron microscopy (TEM) of the soluble particles was performed using a LEO 906E instrument (Zeiss, Oberkochen, Germany) on freeze-dried solutions diluted ten-fold just before freezing. The solid products were dispersed in hexane and drops of the solution were placed on formvar film-coated copper grids. Scanning electron microscopy (SEM) of the precipitates was performed using FEI Quanta 200 instruments (Thermo Fisher Scientific, Waltham, MA, USA). The samples were placed on double-sided sticky carbon tape mounted on aluminum sample holders and then sputter coated with gold using a Balzers SDC 004 Sputter coater (Oerlikon Corporate Pfaffikon, Altendorf, Switzerland). The coating settings (working distance 50 mm, current 15 mA, time 75 s) corresponded to a 12-nm gold coating as per the device manual. The surface area of the composites was measured by nitrogen adsorption at the boiling point of nitrogen (−196 °C) with a Sorbtometr-M device (JSC Katakon, Novosibirsk, Russia), and the data were treated with the Brunauer-Emmett-Teller method [19].

### 2.3. Synthesis of Poly(acryloyl chloride) (PAC)

PAC was synthesized similar to the protocol described earlier [11] by polymerization of acryloyl chloride (5 g) in 20 mL of dioxane with the addition of 0.1 g AIBN in argon atmosphere at 60 °C for 48 h. PAC was used in the reaction with polyamines without comprehensive purification (see below). With the objective to estimate yield and polymerization degree of the PAC, the reaction mixture was poured into water (50 mL) and dialyzed against water. After freeze drying, poly(acrylic acid) was obtained with 90% yield. According to viscometry data, [20] the polymerization degree of the poly(acrylic acid) and, correspondingly of PAC, was found to be 220.

### 2.4. Reaction of PAC with Polyamines

PAC solution in dioxane was poured into 40 mL of hexane in a centrifuge tube where the obtained precipitate was separated by centrifugation (3000× *g*, 5 min) and dissolved in 10 mL of DMFA. The obtained PAC solution was cooled to 0 °C and polyamine in 10 mL of DMFA (cooled to 0 °C) was added. The polyamine:PAC ratios used are presented in Table 1. The reaction mixture was stirred for 30 min at 0 °C and 30 min at room temperature, following which the reaction mixture was poured into 50 mL of water and stirred until the polymer completely dissolved. In experiments with polyamine deficiency, NaOH pellets were added until dissolution. The obtained solution was dialyzed against water and freeze dried. Yields of the polymers were found to be above 95%.

### 2.5. Study of Silicic Acid Condensation in the Presence of New Polymers and Synthesis of Soluble and Solid Composites

In the first stage, the condensation of silicic acid in the presence of new polymeric amines and ampholytes was studied by potentiometry titration of the sodium silicate and polymer mixtures. The obtained results (Table 1) allowed us to select pH ranges where the formation of soluble or insoluble products were expected. Experiments at the desired pH values were performed by mixing stock Na_2_SiO_3_·5H_2_O and polymer solutions (100 mM and 2 g∙L^−1^ correspondingly), the desired amount of water was added, and the pH was adjusted with 1 and 0.1 M HCl solutions. The addition of HCl was done in <1 min using data from preliminary experiments. This allowed us to prevent Si(OH)_4_ condensation at a pH higher than the desired value. Fifty mM HEPES buffer (pH = 7.4) was presented in the solutions prepared for study interactions with oligonucleotide. The obtained solutions were studied with dynamic light scattering (DLS), molybdenum blue assay [21,22], and TEM after freeze-drying. Composite precipitates obtained in some systems were collected with centrifuge (3000× *g*, 10 min), washed twice with cold water (2–4 °C), freeze-dried, and studied with FTIR and SEM. 

### 2.6. Synthesis and Electrophoresis of Oligonucleotide Complexes with Polymers and Composite Nanoparticles

The interaction between 21-mer DNA oligonucleotide GATCTCATCAGGGTACTCCTT-6-FAM and the synthesized polymers or composite nanoparticles was investigated by electrophoresis on agarose gel. Complexes were prepared by mixing solutions of polymer (or composite) and oligonucleotide. The samples were incubated at room temperature for 30 min and placed in the wells of 1% agarose gel. Controls for free oligonucleotide and free polymer (or composite) were also loaded to the gel. The gel running buffer was composed of 40 mM Tris acetate (pH adjusted to pH 7.4) and 1 mM ethylenediaminetetraacetic acid (EDTA). A glycerol gel loading buffer was applied (0.5% sodium dodecyl sulfate, 0.1 M EDTA (pH = 7.4), 50% glycerol for 10× reagent). A solution of 0.05% bromophenol blue was applied to visualize the “dye front” and to calculate the relative mobility (*R*_f_). The gel was run at 90 V and the fluorescein-tagged oligonucleotide was visualized on a UV transilluminator. Mobility of the free DNA oligonucleotide was equal to the mobility of bromophenol blue (*R*_f_ = 1).

### 2.7. Study of Oligonucleotide Complex Penetration into Model Yeast Cells

The cells of *S. cerevisiae* were maintained at 30 °C on YEPD medium (0.5% yeast extract, 1% peptone, 2% glucose). The cells were grown at 30 °C in 10 mL plastic vials with 2 mL of liquid YEPD. Cells in the logarithmic or stationary growth phase were used in the experiments. Oligonucleotide complexes were added to the cell culture and observations were done after 24 h of cultivation. A Motic АЕ-31Т microscope (Motic, Xiamen, China) equipped with a fluorescence attachment (emission at 470 nm) and Moticam Pro 205A camera (Motic, Xiamen, China)) was used for observation of the yeast cells.

## 3. Results

The reaction of PAC with polyamines gave rise to white powder-like products. New polymers were obtained with high yields (>95%), as opposed to similar reactions with long-chain polyamines [23], which were complicated by cross-linking due to the presence of two –NH groups in the polyamine molecules. All polymers (except for P02 and P03) were soluble in water and insoluble in organic solvents. P02 and P03 were soluble in water with HCl acidification to pH 3. The FTIR spectra of the polymers (Figure S1 in Supplementary Materials) revealed bands characteristic of amides (1630 cm^−1^, ν C=O), amines (1150, 1050 cm^−1^, ν C–N; broad absorbance band at 2200–3000 cm^−1^, ν N–H in protonated groups), carboxylic groups (1320 cm^−1^, ν C–O; 1558 cm^−1^, –COO^−^, ν^a^; 1710–1720 cm^−1^, –COOH, ν C=O), and methyl and methylene groups (1460–1470 cm^−1^, δ^a^; 2760-2930 cm^−1^, ν) [24,25]. According to the FTIR spectra, carboxylic units were present in the form of –COOH (P03, P13, and P14 samples), or mainly as carboxylate anions (P04 and P24). The latter samples were prepared with the addition of NaOH during the hydrolysis of –COCl moieties and further purification. These polymers contained non-neutralized amine groups, which was confirmed with the absence of a broad absorbance band at 2200–3000 cm^−1^. The FTIR spectrum of P23 contained a weak band of the –COOH group at 1710 cm^−1^, shoulder at 1570 cm^−1^ (–COO^−^), and band of protonated amines (2200–3000 cm^−1^), which corresponded to the zwitterionic structure of the polymer. 

**Figure 3:**
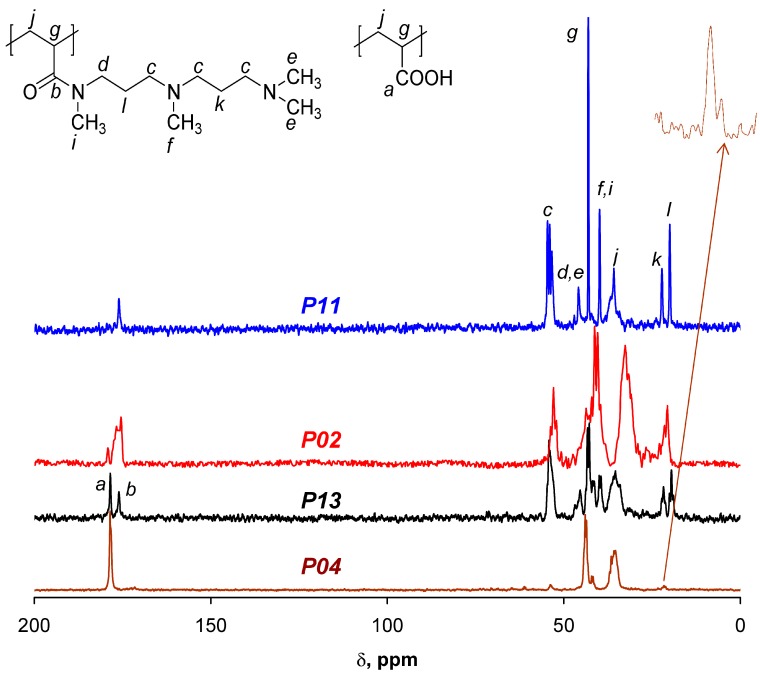
Typical ^13^C NMR spectra of polymeric amine (P11) and ampholytes (P02, P13, and P04) obtained by the reaction between poly (acryloyl chloride) and polyamine.

^1^H NMR spectra of the polymers were not informative and the composition of the copolymers was calculated on the basis of ^13^C NMR data (Figure 3) using integrals of the amide peak near 176 ppm (b) and the peak for carboxylic group near 178.5 ppm (a). In the case of low content of the amide units (Figure 3, P04 sample) the amide peak fused with the carboxylic signal and the peak of the methylene groups near 21 ppm was applied for the calculations. The composition of the synthesized polymers is presented in Table 1 and these data demonstrated that polymeric amines and carboxyl containing polyampholytes could be obtained by the reaction between PAC and polyamines. Molecular weight of the new polymers (*P*_n_) is 26–58 kDa and increases with increase of the length of the polyamine substituent (Table 1). The polydispersity (PDI) of the polymers is typical for macromolecules obtained by radical polymerization.

Copolymers containing a high amount of carboxylic groups are not water soluble at some pH values (Table 1). This is a typical characteristic of polyampholytes as interaction between oppositely-charged functional groups decrease their hydrophilicity. Interestingly, the P23 sample was soluble in the entire pH range studied (2.8–11) as opposed to the P03 and P13 samples, which contained a similar amount of polyamine chains. The high solubility of P23 was correlated to a higher length of the polyamine chains. The –COOH: amine group ratio was found to be 40:60, 36:124, and 46:162 for the P03, P13, and P23 samples, respectively. Potentiometric titration of homopolymers (P01 and P11 samples) was performed to obtain the dependence of *pK* of conjugated acid on ionization degree α, which was calculated according to Reference [26] by Equation (1):

α = ([NaOH] + [H^+^] − [OH^−^])/*C*_total_, (1)

where [NaOH] is the concentration of alkali added; and *C*_total_ is the total concentration of the conjugated acid units. This equation accounted for the self-ionization and hydrolysis of basic and protonated groups. The polymers under investigation contained some amine units associated with HCl. Hence, an excess of HCl was added to the solutions before titration. During NaOH addition, we observed an inflection on the pH vs. volume curve which corresponded to the commencement of titration of the conjugated acid (protonated amine groups). [NaOH] used for α calculations was calculated from this inflection point. *pK* was calculated according to the Henderson-Hasselbach equation [27]:

*pK* = pH − log(α/(1 − α)) (2)

**Figure 4:**
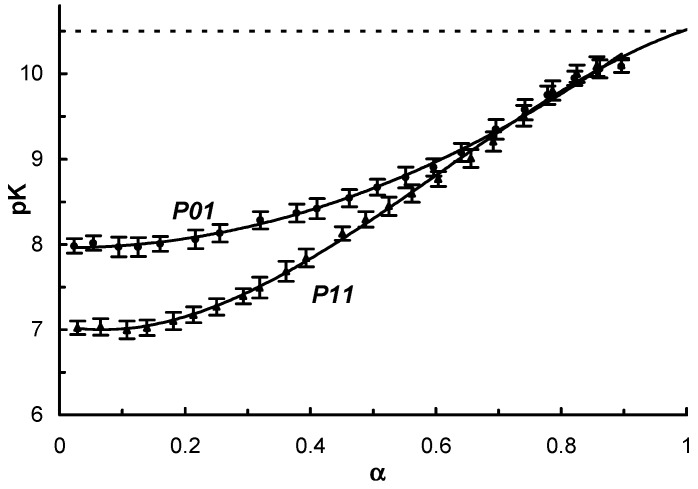
Dependence of *pK* vs. α for P01 and P11 polymers.

**Figure 5:**
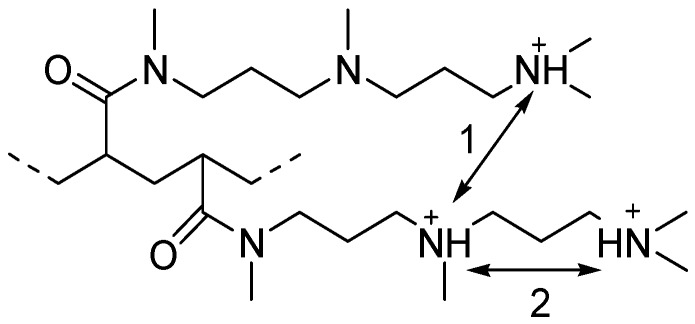
Scheme of electrostatic interactions in the P11 polymer.

The obtained *pK* vs. α data (Figure 4) showed a progressive increase of *pK* with α for P01 and P11 polymers. This effect is typical for polyelectrolytes, e.g., poly(vinyl amine) [28] and arises due to the electrostatic effect. An increase in α corresponded to a decrease in the positive charge on the polymer chain, which decreases the dissociation activity of –NH^+^ groups, thereby increasing the *pK* value. Both curves exhibited an extrapolated *pK* = 10.5 at α = 1, which corresponded to the non-protonated polymeric chain. This value was in agreement with the *pK* of low-molecular amines [29]. In the case of the P11 polymer, two kinds of electrostatic interactions between protonated amine groups were possible (Figure 5): the interactions between neighboring polymeric units (1); and interactions between groups in the same side chain (2).

The initial part of the P11 curve lies significantly below the P01 curve, which points to the higher importance of the interactions in the side chains. Both curves had an initial plateau which corresponded to the elimination of protons from the polymeric chain without a decrease in the strength of the conjugate acid. This effect was possibly connected with a relatively high distance between nitrogen atoms in the neighboring units, and it was necessary to remove some protons for initiating the electrostatic effects. The plateau region observed in the case of P11 was approximately two times shorter when compared to P01 as P11 contained two amine groups in the side chain and an equal α corresponded to twice the number of ionized side chains in P11.

**Figure 6:**
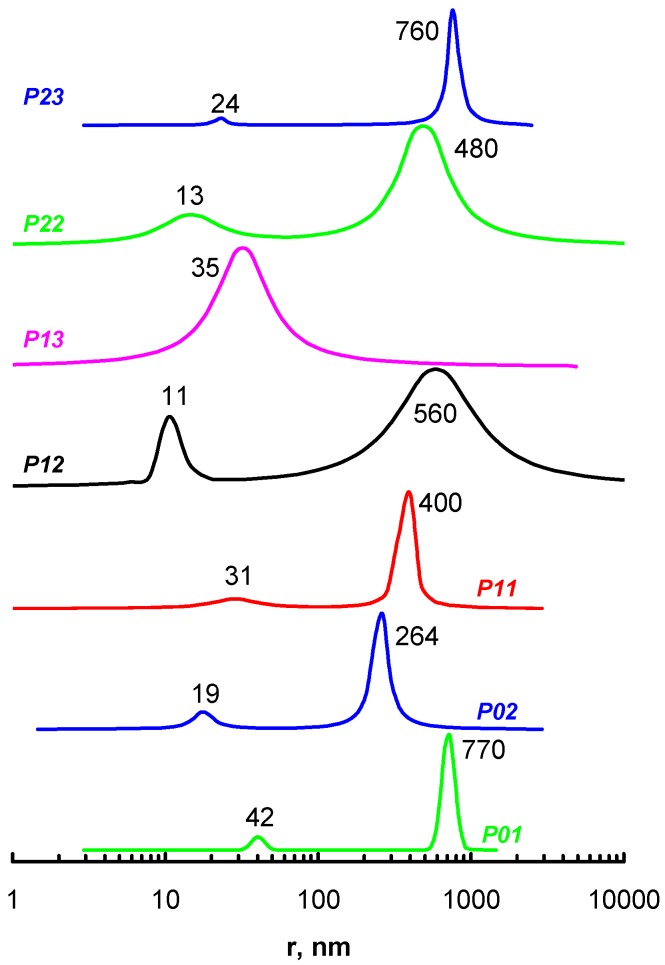
Dynamic light scattering (DLS) data for aqueous solutions of amine-containing polymers at pH 7.4 and a concentration of 2 g∙L^−1^. The polymer abbreviations and peak positions are indicated near the curves.

The particle size of the solutions of the new polymers was studied using DLS (Figure 6) at pH 7.4. Most of the polymers exhibited a bimodal distribution of the particle size: one centered around 10–40 nm, possibly due to single macromolecules or small aggregates; and another at 200–1000 nm due to the formation of large aggregates. The size of the aggregates was often larger than the diameter of the filter pores (450 nm), which pointed to a reversible destruction of the aggregates during filtration, similar to imidazole-containing polyampholytes [30]. The DLS data are presented as time correlation function intensity vs. particle size, which provided an overestimation of the large particles fraction as the scattering intensity is proportional to particle size in degree 3, or more [31].

Polymeric amines have been extensively investigated for their ability to influence silicic acid condensation and in the formation of organo-silica composites. The major interest in this class of molecules stems from the fact that polymeric amines are considered as synthetic models of biogenic molecules, such as silaffins, and the resulting composites formed by them with silicic acid often show interesting and useful properties [32]. We found that titration of the polymer-sodium silicate mixture with HCl resulted in the formation of either turbid or transparent systems (Table 1). Silicic acid is capable of condensing to give rise to poly(silicic acid) (PSA) when pH decreased to 10–11 [20,33] and poly(silicic acid) (PSA) could interact with the polymeric amines. Polymers with high contents of amine groups (P01, P11, and P22) did not show precipitates in the presence of sodium silicate. The introduction of small and moderate amounts of carboxylic units resulted in turbid systems even if the polymer was soluble in the entire pH range being studied. These results may be attributed to the formation of an inter-polymeric complex in the polymer-PSA system. Precipitation of these systems often depends on the acid-base ratio and an excess of amine units can result in soluble non-stoichiometric complexes as discussed in our review [32]. Introduction of acidic carboxylic units into the polymer chain equalized the acid-base ratio and the polymer–PSA complex became insoluble. The polymer-PSA complexes based on polymers with a large amount of acidic units (P04, P14, and P24) were more soluble than the free polymers. We hypothesized that these complexes contained an excess of acidic groups (carboxylic and silanol) which made them soluble.

**Figure 7:**
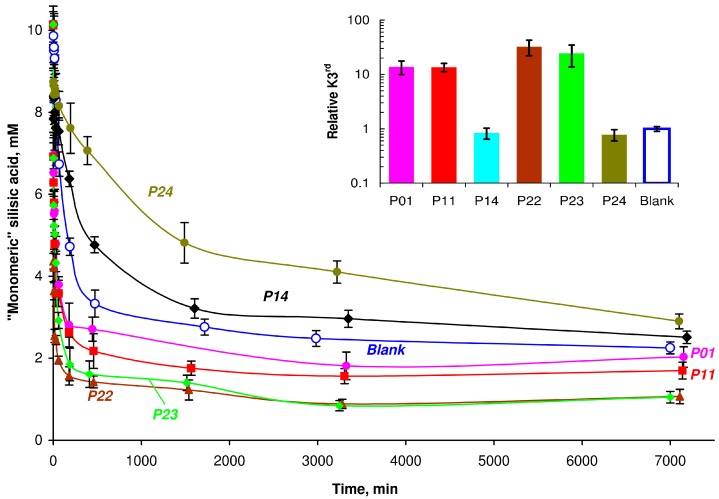
Effect of the new copolymers on condensation of silicic acid at pH 7. The *k*_third_ constants for the rate of Si(OH)_4_ condensation at initial stage (0–60 min) are relative to the blank. Initial Si(OH)_4_ concentration is 10 mM, polymers = 1.5 g∙L^−1^.

The influence of various substances on the condensation of silicic acid can be monitored using the molybdate method [21,22], which allowed the measurement of the monomer and dimer concentrations of silicic acid. Study of Si(OH)_4_ condensation in the presence of new polymers (Figure 7) revealed an acceleration of the condensation process with polymers containing a considerable amount of amine units similar to our results with poly(vinyl amine) [34]. The effect was visible at the very early stages of condensation (*k*_third_ values) and the polymers decreased the equilibrium concentration of free silicic acid in the later stages. Polymers containing an excess of carboxylic units (P14 and P24) did not influence condensation during the early stages, and inhibited condensation at the hour and day time intervals. Condensation of silicic acid at pH 7 proceeded mainly as an S_N_2 reaction between the –Si–OH and –Si–O^−^ moieties [33]. Silanol anions arose from the primary PSA particles as Si(OH)_4_ is a very weak acid (*pK* = 9–10) [21,32] in comparison to PSA (*pK*_0_ = 6–7). Interaction between PSA (including primary oligomers) and polyamine chain increased the amount of silanol anions (–SiO^− +^HN–) and an acceleration in condensation was observed. In the case of the P14 and P24 polymers, the macromolecular chains were negatively charged at pH 7 due to an excess of carboxylic units that prevented their interaction with primary siliceous oligomers, and these polymers did not influence condensation in early stages. Inhibition of condensation at the later time points resulted in a decrease of silanol units in the system. A similar effect was observed in the presence of poly(ethylene glycol) [35], poly(1-vinylpyrrolidone) [36], and poly(1-vinylimidazole) [37]. These polymers form hydrogen bonds with silanol units which stabilize non-ionized –Si-OH groups, thus decreasing the condensation rate. P14 and P24 polymers contain carboxylic anions at pH 7 and these groups can be treated as weak bases (*pK* of the conjugated acid is 6–6.5 at pH 7) [38]. We hypothesized that the carboxylic anions could stabilize non-ionized –Si–OH groups by means of hydrogen bonding (–C(O)O^−^∙∙∙H–O–Si–) and these interactions resulted in the inhibition of condensation. Possible spectroscopic evidence of these bonds was reported by Danilovtseva et al. [39].

**Figure 8:**
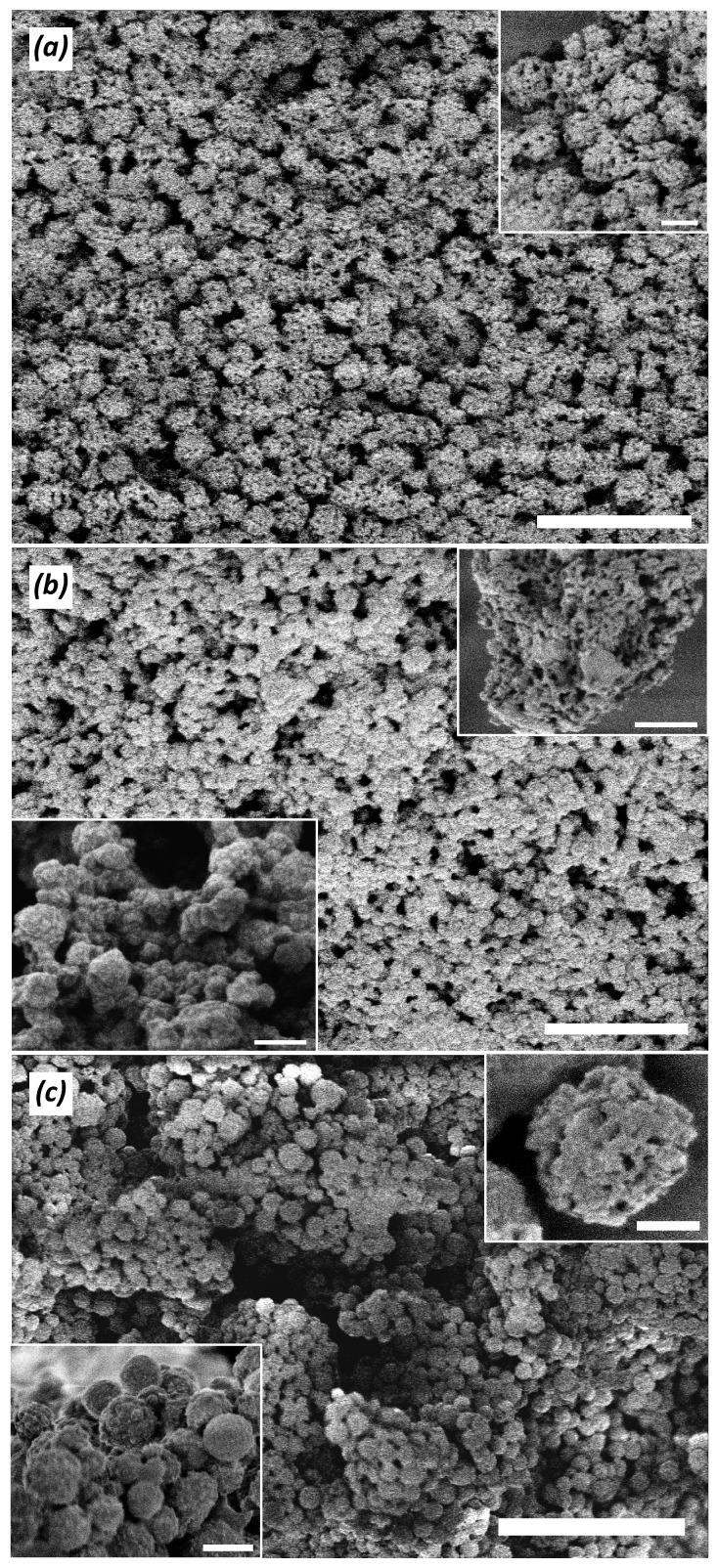
Scanning electron microscopy (SEM) images of composite precipitates obtained under silicic acid condensation in the presence of new copolymers. (**a**) P02; (**b**) P03; and (**c**) P13 copolymers. Initial Si(OH)_4_ concentration is 10 mM, polymers = 1.5 g∙L^−1^, pH = 7, condensation time = 24 h. Scale bar represents 1 µm (**a**,**b**), 200 nm (insertion in (**a**), bottom insertion in (**b**), top insertion in (**c**)), 500 nm (top insertion in (**b**) and bottom insertion in (**c**), and 5 µm (**c**)).

Composite precipitates were obtained when silicic acid was condensed in the presence of polymers P02, P03, and P13 at pH 7. The precipitates contained silica and organic polymers according to the FTIR spectra Si–O–Si (1070 cm^−1^) and Si–OH (965 cm^−1^) bands, (Figure S2 in Supplementary Materials). The composite particles appeared as 200–500 nm (in diameter) spheres decorated with small particles about 50 nm (Figure 8). BET surface area for composites based on P02, P03, and P13 polymers was 5.8, 1.8, and 23.1 m^2^∙g^−1^, respectively. These values were considerably lower than those reported for silica precipitated in aqueous medium (several hundred m^2^∙g^−1^). This anomaly can be attributed to the coating of the siliceous nanoparticles with an organic polymer that led to a decrease in the formation of micro- and mesopores.

**Figure 9:**
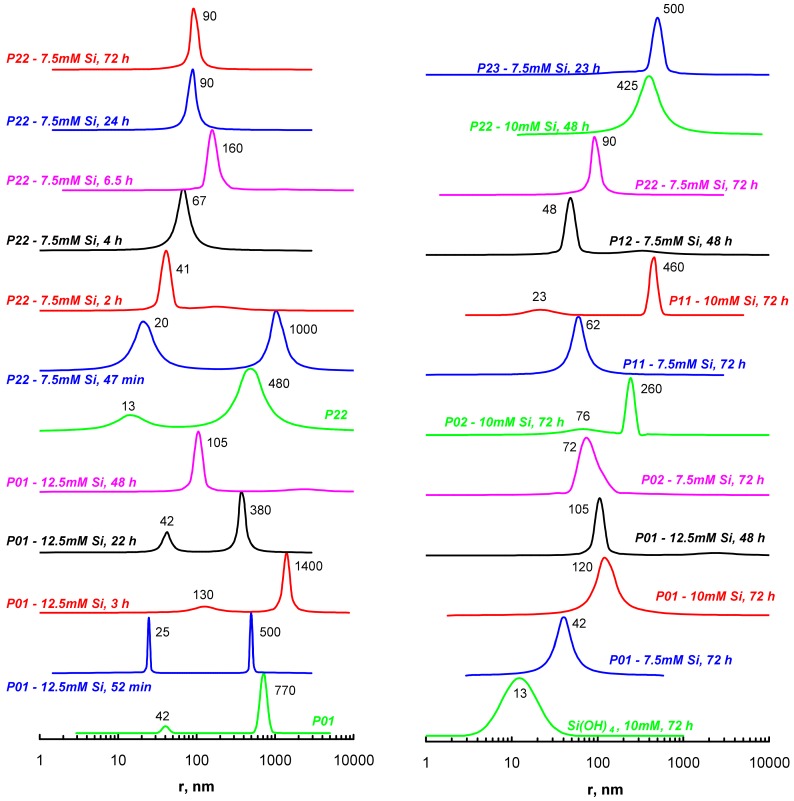
Dynamic light scattering (DLS) data for aqueous solutions of composite particles obtained under silicic acid condensation in the presence of new polymers (0.4 g∙L^−1^) at pH 7.4. Initial silicic acid concentrations, the condensation time, and peak positions are indicated near the curves. The left curves show the typical behavior of particle size values during condensation and the right curves represent the DLS of the resulting composite nanoparticles. Some systems had precipitates after 72 h of the reaction and in these cases earlier data are presented.

**Figure 10:**
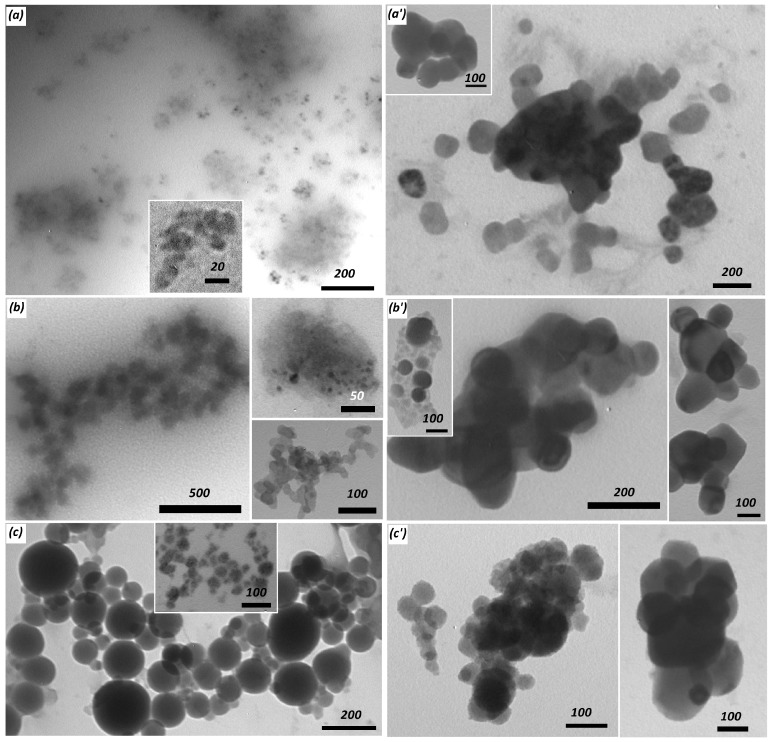
Transmission electron microscopy (TEM) images of composite particles obtained under silicic acid condensation in the presence of new polymers. (**a**) P01; (**b**) P11; and (**c**) P22 polymers. (**a’**,**b’**,**c’**) complexes of the respective composite particles and DNA oligonucleotide. Initial Si(OH)_4_ concentration is 7.5 mM, polymers = 0.4 g∙L^−1^, and composite = 10 μM DNA ratio is 4:1 (volume to volume). Scale bars are presented in nm.

Condensation of silicic acid in the presence of polymeric amines often results in composite nanoparticles that are stable in aqueous solutions [32,40]. We studied the formation of the composite nanoparticles at a decreased polymer concentration (0.4 g∙L^−1^) with the objective to obtain soluble products with a high silica content. We found that the stable polymer–PSA solutions were obtained at silicic acid concentrations of 7.5–12.5 mM at pH 7.4. DLS data (Figure 9) showed that free silicic acid formed approximately 20 nm (in diameter) particles after three days of condensation. Reactions in the presence of polymers proceeded through bimodal distribution of the particle size and after 1–2 days, uniformly distributed particles were observed. The size of the resulting particles increased with an increase of Si(OH)_4_ concentration and with the introduction of carboxylic groups in the polymeric chain. Freeze-dried solutions of the composite nanoparticles were studied with transmission electron microscopy (TEM, Figure 10). The obtained material contained electron dense particles with sizes comparable to the size of the smallest silica particles (Figure 9). The TEM images also revealed that the silica particles were surrounded by a transparent layer, probably due to the organic polymer chains. The size of the particles from the TEM data was several times lower than that obtained with DLS, possibly due to the association of the particles in an aqueous medium. Moreover, DLS recorded the hydrodynamic radius of the particles and hence was always greater than the size observed in electron microscopy. 

Polymeric amines have been widely investigated as gene transfer agents [41,42]. The positive charge of the polyamine chain provides effective binding with nucleic acid. The obtained complex particles internalize in the cells via endocytosis and end up in the endosomes (small membrane-bound compartments). The endosomes are transported to lysosomes (organelles containing hydrolytic enzymes) which degrade a wide range of biomacromolecules. The polyamine coating must aid the complexed nucleic acid to escape lysosomal degradation. Earlier reports have demonstrated that polyethylenimine (PEI) is an effective transfection agent as it enables the destruction of endosomes before approaching lysosome [43]. This effect was explained based on the ability of PEI to work as a “proton sponge”, therefore, showing a buffer capacity from the physiological pH to acid range [44]. Proton pumping into the endosome and a corresponding influx of chloride ions results in an increase of ionic strength inside the endosome. This is accompanied by osmotic swelling, which gives rise to the endosome destruction and release of the nucleic acid cargo into cytosol. Thus, synthesis of polymers for gene delivery is aimed on creation of structures showing a high buffer capacity in the neutral and slightly acidic range. Complex polymer structures containing high amounts of tertiary amine groups were recently obtained [45,46,47,48] and showed a high buffer capacity combined with enhanced transfection activity.

Buffer capacity of the new polymers (Table 2) was calculated from the potentiometry data according to the International Union of Pure and Applied Chemistry (IUPAC) recommendations as a derivative of the added NaOH concentration vs. pH (the number of moles of strong base required to change the pH by one unit when added to one liter of the solution) [49]. Polymers containing one amine group in the side chain (P01 and P02) showed a drastic decrease of the buffer capacity in slightly acidic pH when compared with polymers whose side chains contained two or three amine moieties. This effect was connected with a decrease of basicity of neighboring amine groups after the protonation of one nitrogen atom in the side chain. The buffer capacity of the ampholyte polymer (P23) did not depend on pH in the studied interval as the carboxylate anion was also a basic unit in addition to the amine groups. Thus, polymers with two or three amine moieties in the side chains are more promising agents for gene delivery according to the proton sponge theory. Polyampholyte P23 is also suitable to study in gene delivery as its average unit charge is +0.43 at pH 7.4 when calculated as per Annenkov et al. [30] and this polymer is capable of interacting with negatively charged nucleic acids.

**Figure 11:**
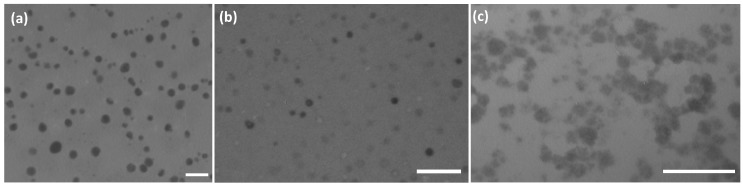
TEM images of polymer-oligonucleotide complexes. The sample compositions corresponds to complexes in line 7 ((**a**), P11 polymer), line 11 ((**b**), P12), and 19 ((**c**), P23) from Table 3. Scale bar represents 200 (**a**,**b**), and 100 (**c**) nm.

The ability of new polymers to interact with nucleic acids was verified with 21-mer DNA. Gel electrophoresis data (Table 3, Figures S3–S5 in the Supplementary Materials) showed that polymers with a high content of amine groups (P01, P02, P11, P12, P22, and P23, #1, 2, 5–7, 10, 16, and 18 in Table 3) gave positively-charged complexes with the oligonucleotide. Decreases in the amine content in the copolymer resulted in an almost neutral complex (P13), or the complex was absent (P03 and P04). The polymer:oligonucleotide ratio could change the charge of the complex from positive to neutral (Table 3, #11 and 12, Figures S4 and S5 in the Supplementary Materials). Furthermore, it was observed that a lower concentration of the polymer (0.2–0.3 g/L, Table 3, #8 ,9, 13, and 14) resulted in incomplete complexation, as evident from the spreading electrophoretic blots from *R*_f_ = 0 to near 1. ζ-Potential of the polymer-oligonucleotide complexes is positive in the case of systems which show full complexing: 20.3, 14.9, 5.8, 15.7, and 9.1 mV for lines 7, 11, 12, 15, and 19 from Table 3. The size of the complex particles is below 50 nm according to TEM data (Figure 11).

The structure and charge of the oligonucleotide-containing complexes are important factors that influence the transfection efficiency of the oligonucleotide. Various molecules have been investigated in this area and one approach is to use siliceous composites [4,5]. We studied the ability of composite siliceous nanoparticles based on new polymeric amines to interact with the oligonucleotide (Table 4, Figures S6 and S7 in the Supplementary Materials) and found the following:

- the presence of PSA in the system effectively shifted the charge of the particles from positive in the case of free polymers to neutral and negative (Table 4, #2–5, 7, 13–17 compared with #8–11 and 18–19);
- high content of PSA in the composite decreased or prevented interaction with the oligonucleotide (Table 4, #1–3, 6, and 12) which became apparent in the DNA fluorescence near *R*_f_ = 1.

Thus, the introduction of carboxylic units into a polyamine chain and/or association with PSA allowed us to control the charge of the DNA-containing aggregates. TEM data (Figure 10) showed that composite DNA particles were below 200 nm in diameter. The addition of DNA to siliceous composite nanoparticles resulted in the aggregation of small particles based on P01 and P02 polymers, while denser particles from the P22 polymer persisted with their shape in the presence of DNA. Slightly positive systems are the most promising as gene delivery agents, and were obtained with P01, P11, P12, P13, P22, and P23 polymers (Table 3, #11, 15, 19, Table 4, #5 and 16–20).

**Figure 12:**
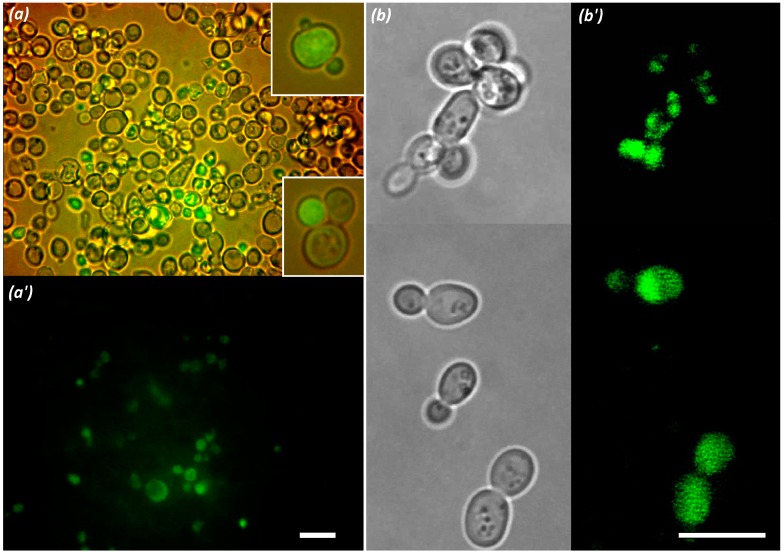
Images of *S. cerevisiae* cells after incubation with oligonucleotide-complexed polymer P23 (**a**,**a’**), P22 (insertions in (**a**)), and with oligonucleotide-complexed composite based on P01 polymer (**b**,**b’**). Image (**a**) and insertion images are obtained by combining light illumination and fluorescent excitation, (**b**) shows light images, and (**a**’,**b**’) show fluorescent images. Green fluorescence originates from fluorescent-tagged oligonucleotide. Composition of the complexes corresponds to line 19 in Table 3 (**a**,**a**’), line 20 in Table 4 (insertions in (**a**)), and line 4 in Table 4 (**b**,**b**’). Complex: cell culture ratio was 1:3. Scale bars represents 10 μm.

Short DNA and RNA chains are not so active in complexing with polymers traditionally applied with plasmid DNA ([50] and references in this review). This was explained with the absence of spatial flexibility of the short chains which results in high constraints on the formation of cooperative interactions with the polymer. Amine groups in the polymers obtained in this work are connected to the main chain through amide and trimethylene spacers, which decrease the entropy loss during complexing and allows the formation of stable polymer-oligonucleotide complexes. The ability of the new polymers and composite nanoparticles to facilitate penetration of oligonucleotides into living cells was demonstrated with *S. cerevisiae* yeast cells (Figure 12). The appearance of green fluorescence inside cells is evidence of the penetration of the oligonucleotide complex.

## 4. Conclusions

We synthesized a set of homopolymers and copolymers starting from poly(acryloyl chloride) and short-chain polyamines. The polymers were obtained by polymer-analogous reactions under moderate conditions, which prevented the destruction of the main chain and provided a set of polymers with highly variable functionality and similar polymerization degrees. New polymeric amines and ampholytes were active in interaction with poly(silicic acid) and oligonucleotides, which allowed the design of composite nanoparticles, ordered composite materials, and oligonucleotide complexes for gene delivery.

## Figures and Tables

**Figure 2:**
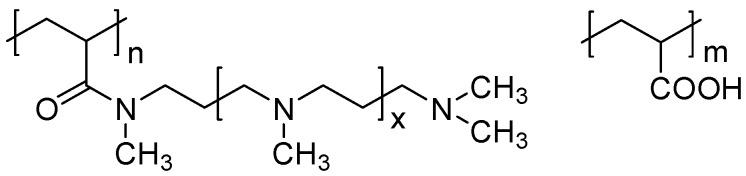
Structure of the synthesized polymers.

**Table 1:** Composition of the reaction mixture and polymers obtained by PAC reaction with polyamines.

Polymer #	^1^ x	Amine:–COCl Initial Ratio	^1^ *n*:*m*	*M*_n_, kDa	*M*_w_, kDa	PDI = *M*_w_/*M*_n_	pH Interval of Heterogeneity	
							^2^ Polymer	^3^ Polymer and Si(OH)_4_
P01	0	1.5:1	100:0	30.0	41.0	1.37	-	-
P02	0	1:1	98:2	25.8	37.9	1.47	-	2.8–9.8
P03	0	0.5:1	40:60				2.7–9.7	2.8–9.8
P04	0	0.1:1	7:93				2.8–7.3	-
P11	1	1.5:1	100:0	44.4	66.6	1.50	-	-
P12	1	1:1	95:5	44.5	66.7	1.50	-	<10
P13	1	0.5:1	36:62	30.2	47.2	1.56	7.8–9.9	3–10
P14	1	0.1:1	12:88				2.7–10.0	2.2–5.2
P22	2	1:1	90:10	57.7	78.9	1.37	-	-
P23	2	0.5:1	46:54	52.8	83.3	1.58	-	6.2–9.2
P24	2	0.1:1	15:85				5.6–10.0	2.3–5.2

^1^ According to Figure 2. ^2^ Data were obtained by a potentiometry titration of solutions containing 1.5 g∙L^−1^ of polymer after acidification of up to pH 2.6 with 1 M HCl. Titrant = 0.1 M NaOH. The titration was done up to pH 11.5; “-” refers to the solubility in the entire pH range (2.6–11.5) studied. ^3^ Data were obtained by a potentiometry titration of solutions containing 10 mM of Na_2_SiO_3_ and 1.5 g∙L^−1^ of polymer. Titrant = 0.1 M HCl. The titration was done up to pH 2.2; “-” refers to the solubility in the entire pH range (2.2–11.5) studied.

**Table 2:** Buffer capacity (mmol∙L^−1^) of amine-containing polymers (1.5 g∙L^−1^ solutions).

Polymer	pH				
	7.4	7	6.5	6	5.5
P01	1.6	1.3	0.8	0.4	0.06
P02	1.2	0.9	0.6	0.3	0.1
P11	1.7	1.8	1.8	1.5	1.0
P12	1.8	1.7	1.5	1.2	0.9
P22	1.5	1.5	1.5	1.3	1.2
P23	0.9	0.9	0.9	0.9	0.9

**Table 3:** Gel electrophoresis results for DNA oligonucleotide complex with new polymers.

#	Polymer	Amine Units, mol %	^1^ Polymer Conc., g∙L^−1^	^2^ R_f_
Free DNA Oligonucleotide				1
1	P01	100	2	−0.59–0
2	P02	98	2	−0.43–0
3	P03	40	2	1
4	P04	7	2	1
5	P11	100	2	−0.41–−0.03
6	P11	100	1	−0.20–−0.04
7	P11	100	0.7	−0.22–0
8	P11	100	0.3	0; 0.85
9	P11	100	0.2	0; 0–0.86
10	P12	95	2	−0.25–−0.03
11	P12	95	1	−0.08–0
12	P12	95	0.7	0
13	P12	95	0.3	0–0.93
14	P12	95	0.2	0–0.96
15	P13	36	2	−0.13–0
16	P22	90	2	−0.54–0
17	P22	90	1	−0.08–−0.02
18	P23	46	2	−0.45–0
19	P23	46	1	−0.06–−0.02
20	P24	12	2	1

^1^ DNA oligonucleotide was applied as 10 µM solution. Polymer:oligonucleotide ratio (volume to volume) was 2:1. ^2^ Negative *R*_f_ values correspond to substance movement towards the negative electrode. Interval of *R*_f_ values corresponds to spreading electrophoretic blots.

**Table 4:** Gel electrophoresis results for DNA oligonucleotide complex with new polymers and composites.

#	Polymer	Amine Units, mol %	^1^ Silicate Conc., g∙L^−1^	Polymer (Composite): DNA Ratio	^2^ *R*_f_
1	P01	100	12.5	2:1	1
2	P01	100	10	2:1	0–0.95
3	P01	100	10	4:1	0.33–1
4	P01	100	7.5	2:1	0
5	P01	100	7.5	4:1	0; 0–0.15
6	P02	98	10	2:1	0.87–1
7	P02	98	7.5	4:1	0.22–0.719
8	P01	100	Free polymer	2:1	−0.35–−0.03
9	P01	100	Free polymer	4:1	−0.39–−0.03
10	P02	98	Free polymer	2:1	−0.38–−0.03
11	P02	98	Free polymer	4:1	−0.34–−0.03
12	P11	100	10	4:1	0.77–0.92
13	P11	100	7.5	4:1	0
14	P12	95	7.5	4:1	0
15	P22	90	10	4:1	0
16	P22	90	7.5	4:1	−0.08
17	P23	46	7.5	4:1	−0.06
18	P11	100	Free polymer	4:1	−0.07–−0.03
19	P12	95	Free polymer	4:1	−0.10–−0.03
20	P22	90	Free polymer	4:1	−0.07–−0.03

^1^ DNA oligonucleotide was applied as 10 µM solution. Polymer concentration was 0.4 g∙L^−1^. ^2^ Negative *R*_f_ values correspond to substance movement to the negative electrode. Interval of *R*_f_ values corresponds to spreading electrophoretic blots.

## Supplementary Materials

The following are available online at www.mdpi.com/2073-4360/9/11/624/s1, Figure S1: FTIR spectra of the new polymers; Figure S2: FTIR spectra of new polymers and siliceous composites; Figures S3–S5: Representative gel electrophoresis results for DNA oligonucleotide complex with new polymers; Figures S6 and S7: Representative gel electrophoresis results for DNA oligonucleotide complex with new polymers and composites.

## References

[1] Zhang Y., Broekhuis A.A., Stuart M.C.A., Picchioni F. (2008). Polymeric amines by chemical modifications of alternating aliphatic polyketones. J. Appl. Polym. Sci..

[2] Butun V., Liu S., Weaver J.V.M., Bories-Azeau X., Cai Y., Armes S.P. (2006). A brief review of “schizophrenic” Block copolymers. React. Funct. Polym..

[3] Smedt S.C.D., Demeester J., Hennink W.E. (2000). Cationic polymer based gene delivery systems. Pharm. Res..

[4] Pandit V., Watson A., Ren L., Mixon A., Kotha S.P. (2015). Multilayered nanoparticles for gene delivery used to reprogram human foreskin fibroblasts to neurospheres. Tissue Eng. Part C.

[5] Miyata K., Gouda N., Takemoto H., Oba M., Lee Y., Koyama H., Yamasaki Y., Itake K., Nishiyama N., Kataoka K. (2010). Enhanced transfection with silica-coated polyplexes loading plasmid DNA. Biomaterials.

[6] Nascimento A.V., Singh A., Bousbaa H., Ferreira D., Sarmento B., Amiji M.M. (2014). Mad2 checkpoint gene silencing using epidermal growth factor receptor-targeted chitosan nanoparticles in non-small cell lung cancer model. Mol. Pharm..

[7] Kim Y.-K., Cho C.-S., Cho M.-H., Jiang H.-L. (2014). Spermine-*alt*-poly(ethylene glycol) polyspermine as a safe and efficient aerosol gene carrier for lung cancer therapy. J. Biomed. Mater. Res. Part A.

[8] Scholz C., Kos P., Leclercq L., Jin X., Cottet H., Wagner E. (2014). Correlation of length of linear oligo(ethanamino) amides with gene transfer and cytotoxicity. ChemMedChem.

[9] Allen M.H., Day K.N., Hemp S.T., Long T.E. (2013). Synthesis of folic acid-containing imidazolium copolymers for potential gene delivery applications. Macromol. Chem. Phys..

[10] Gu L., Zhu S., Hrymak A.N.J. (2002). Acidic and basic hydrolysis of poly(*N*-vinylformamide. J. Appl. Polym. Sci..

[11] Buruiana E.C., Buruiana T., Hahui L. (2007). Preparation and characterization of new optically active poly(*N*-acryloyl chloride) functionalized with (*S*)-phenylalanine and pendant pyrene. J. Photochem. Photobiol. A.

[12] Annenkov V.V., Zelinskiy S.N., Danilovtseva E.N., Perry C.C. (2009). Synthesis of biomimetic polyamines. ARKIVOC.

[13] Kroger N., Deutzmann R., Sumper M. (1999). Polycationic peptides from diatom biosilica that direct silica nanosphere formation. Science.

[14] Das A., Theato P. (2016). Activated ester containing polymers: Opportunities and challenges for the design of functional macromolecules. Chem. Rev..

[15] Kakuchi R., Wongsano K., Hoven V.P., Theato P. (2014). Activation of stable polymeric esters by using organo-activated acyl transfer reactions. J. Polym. Sci. Part A.

[16] Yamazaki S., Noda I., Tsutsumi A. (2000). l3C NMR relaxation of poly(acrylic acid) in aqueous solution. Effects of charge density on local chain dynamics. Polym. J..

[17] Pavlov G.M., Korneeva E.V., Ebel C., Gavrilova I.I., Nesterova N.A., Panarin E.F. (2004). Hydrodynamic behavior, molecular mass and conformational parameters of poly(vinylformamide) molecules. Polym. Sci. A.

[18] Box M.J. (1965). A new method of constrained optimization and a comparison with other methods. Comput. J..

[19] Brunauer S., Emmett P.H., Teller E. (1938). Adsorption of gases in multimolecular layers. J. Am. Chem. Soc..

[20] Newman S., Krigbaum W.R., Laugier C., Flory P.J. (1954). Molecular dimensions in relation to intrinsic viscositie. J. Polym. Sci..

[21] Iler R. (1979). The Chemistry of Silica.

[22] Belton D., Paine G., Patwardhan S.V., Perry C.C. (2004). Towards an understanding of (bio)silicification: The role of amino acids and lysine oligomers in silicification. J. Mater. Chem..

[23] Annenkov V.V., Pal’shin V.A., Verkhozina O.N., Larina L.I., Danilovtseva E.N. (2015). Composite nanoparticles: A new way to siliceous materials and a model of biosilica synthesis. Mater. Chem. Phys..

[24] Gordon A.J., Ford R.A. (1973). The Chemist’s Companion: A Handbook of Practical Data. Techniques, and References.

[25] Smith A.L. (1979). Applied Infrared Spectroscopy: Fundamentals, Techniques, and Analytical Problem-Solving.

[26] Strauss U.P., Barbieri U.P., Wong G. (1979). Analysis of ionization equilibriums of polyacids in terms of species population distributions. Examination of a “two-state” conformational transition. J. Phys. Chem..

[27] Katchalsky A., Spitnik P. (1947). Potentiometric titrations of polymethacrylic acid. J. Polym. Sci..

[28] Katchalsky A., Mazur J., Spitnik P. (1957). Polybase properties of polyvinylamine. J. Polym. Sci..

[29] Hall H.K. (1957). Correlation of base strengths of amines. J. Am. Chem. Soc..

[30] Annenkov V.V., Danilovtseva E.N., Tenhu H., Aseyev V., Hirvonen S.-P., Mikhaleva A.I. (2004). Copolymers of 1-vinylimidazole and (meth)acrylic acid: Synthesis and polyelectrolyte properties. Eur. Polym. J..

[31] Schärtl W. (2007). Light Scattering from Polymer Solutions and Nanoparticle Dispersions.

[32] Annenkov V.V., Danilovtseva E.N., Pal’shin V.A., Verkhozina O.N., Zelinskiya S.N., Krishnan U.M. (2017). Silicic acid condensation under the influence of water-soluble polymers: From biology to new materials. RSC Adv..

[33] Brinker C.J., Scherer G.W. (1990). Sol-Gel Science: The Physics and Chemistry of Sol-Gel Processing.

[34] Annenkov V.V., Danilovtseva E.N., Pal’shin V.A., Aseyev V.O., Petrov A.K., Kozlov A.S., Patwardhan S.V., Perry C.C. (2011). Poly (vinyl amine)—Silica composite nanoparticles: Models of the silicic acid cytoplasmic pool and as a silica precursor for composite materials formation. Biomacromolecules.

[35] Demadis K.D., Tsistraki A., Popa A., Iliab G., Visa A. (2012). Promiscuous stabilisation behaviour of silicic acid by cationic macromolecules: The case of phosphonium-grafted dicationic ethylene oxide bolaamphiphiles. RSC Adv..

[36] Spinde K., Pachis K., Antonakaki I., Paasch S., Brunner E., Demadis K.D. (2011). Influence of polyamines and related macromolecules on silicic acid polycondensation: Relevance to “soluble silicon pools”?. Chem. Mater..

[37] Annenkov V.V., Danilovtseva E.N., Likhoshway Y.V., Patwardhan S.V., Perry C.C. (2008). Controlled stabilisation of silicic acid below ph 9 using poly(1-vinylimidazole). J. Mater. Chem..

[38] Morlay C., Cromer M., Mouginot Y., Vittori O. (1998). Potentiometric study of Cu(II) and Ni(II) complexation with two high molecular weight poly(acrylic acids). Talanta.

[39] Danilovtseva E.N., Pal’shin V.A., Likhoshway Y.V., Annenkov V.V. (2011). Condensation of silicic acid in the presence of co(1-vinylimidazole–acrylic acid). Adv. Sci. Lett..

[40] Sumper M. (2004). Biomimetic patterning of silica by long-chain polyamines. Angew. Chem. Int. Ed..

[41] Yang J., Liu H., Zhang X. (2014). Design, preparation and application of nucleic acid delivery carriers. Biotechnol. Adv..

[42] Dubruel P., Schacht E. (2006). Vinyl polymers as non-viral gene delivery carriers: Current status and prospects. Macromol. Biosci..

[43] Akinc A., Thomas M., Klibanov A.M., Langer R. (2005). Exploring polyethylenimine-mediated DNA transfection and the proton sponge hypothesis. J. Gene Med..

[44] Behr J.P. (1997). The proton sponge: A trick to enter cells the viruses did not exploit. Chimia.

[45] Zhou D., Cutlar L., Gao Y., Wang W., O’Keeffe-Ahern J., McMahon S., Duarte B., Larcher F., Rodriguez B.J., Greiser U. (2016). The transition from linear to highly branched poly(b-amino ester)s: Branching matters for gene delivery. Sci. Adv..

[46] Gao Y., Huang J.Y., O’Keeffe Ahern J., Cutlar L., Zhou D., Lin F.H., Wang W. (2016). Highly branched poly(β-amino esters) for non-viral gene delivery: High transfection efficiency and low toxicity achieved by increasing molecular weight. Biomacromolecules.

[47] Liu S., Yang J., Ren H., O’Keeffe-Ahern J., Zhou D., Zhou H., Chen J., Guo T. (2016). Multifunctional oligomer incorporation: A potent strategy to enhance the transfection activity of poly(l-lysine). Biomater. Sci..

[48] Zhou D., Gao Y., Aied A., Cutlar L., Igoucheva O., Newland B., Alexeeve V., Greiser U., Uitto J., Wang W. (2016). Highly branched poly(β-amino ester)s for skin gene therapy. J. Control. Release.

[49] Sandeli E.B., West T.S. (1969). Recommended nomenclature for titrimetric analysis. Pure Appl. Chem..

[50] Vader P., van der Aa L.J., Storm G., Schiffelers R.M., Engbersen J.F. (2012). Polymeric carrier systems for siRNA delivery. Curr. Top. Med. Chem..

