# Supplementary material for: Polymeric Amines and Ampholytes Derived from Poly(acryloyl chloride): Synthesis, Influence on Silicic Acid Condensation and Interaction with Nucleic Acid
Source: Polymers (Basel). 2017 Nov 16;9(11):624. doi: 10.3390/polym9110624 (PMC6418922; doi:10.3390/polym9110624)
Supplement: Supplementary file 1 [file polymers-09-00624-s001.pdf]

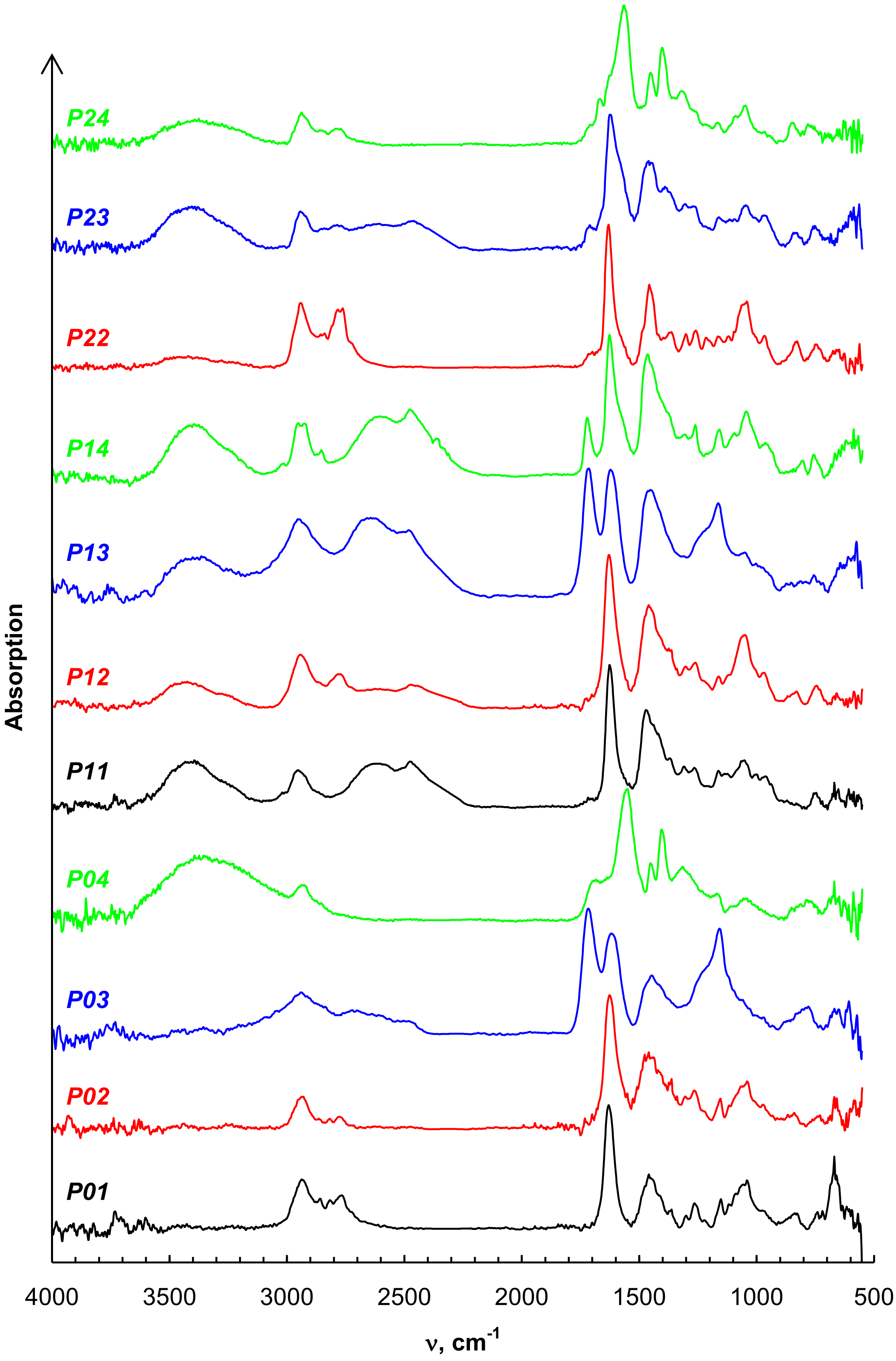

Figure S1. FTIR spectra of the new polymers. The CO_2 band at 2300 cm^{-1} was erased.

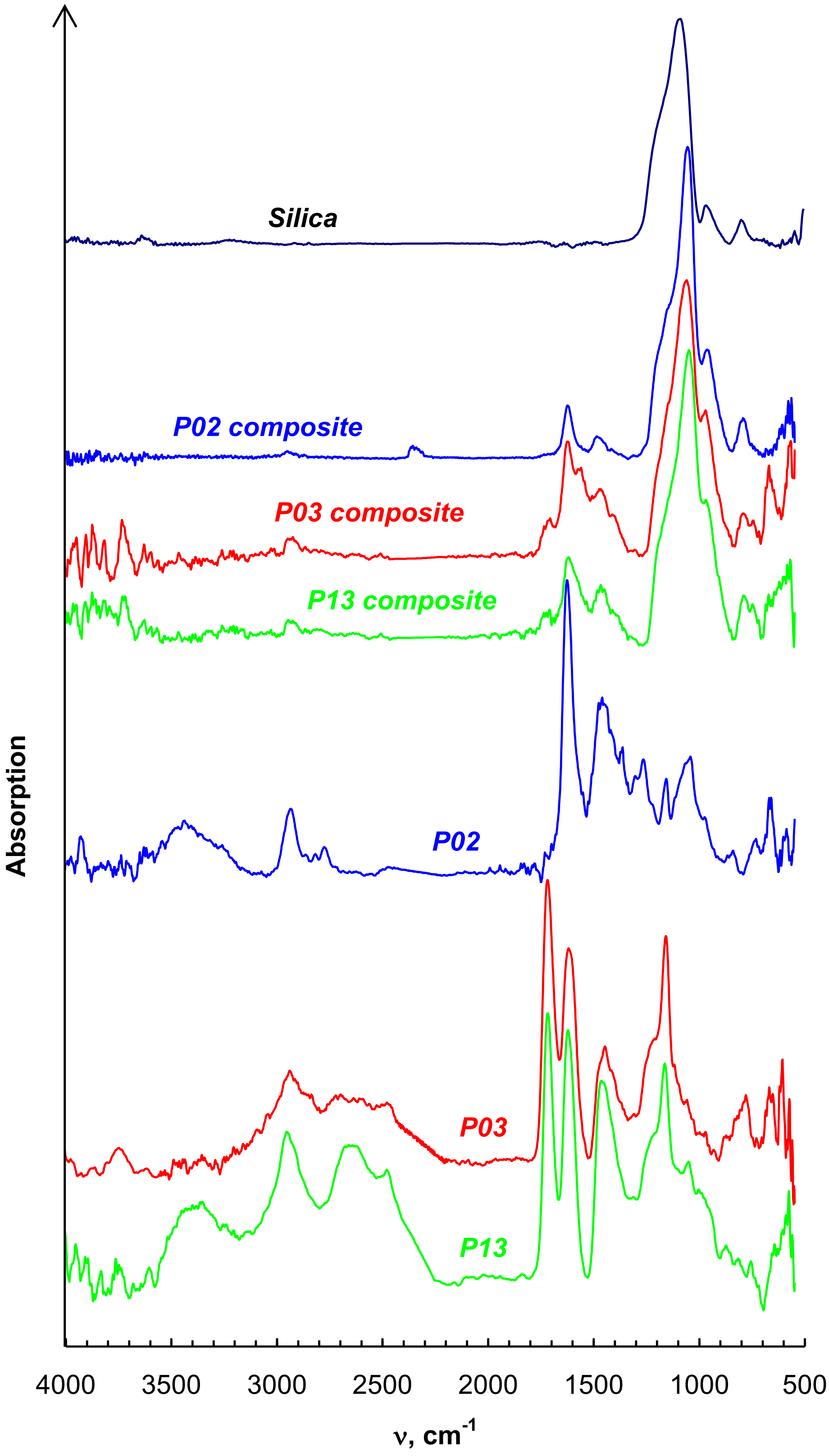

Figure S2. FTIR spectra of new polymers and siliceous composites.

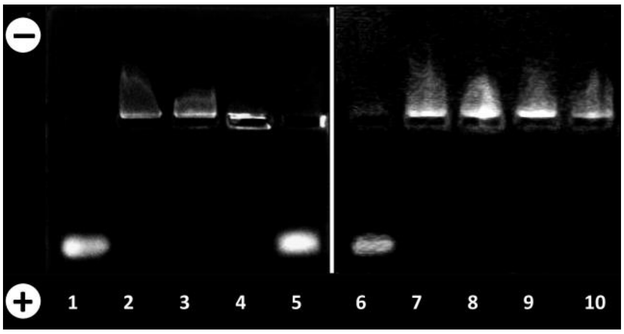

Figure S3. Gel electrophoresis results for DNA oligonucleotide complex with new polymers. Lanes 1, 6: free oligonucleotide; 2: P11; 3: P-12; 4: P13; 5: P04; 7: P01; 8: P02; 9: P22; 10: P23. Concentration of the polymers was 2 g·L^{-1} and oligonucleotide was 10 μM. The polymer:oligonucleotide ratio (volume to volume) was 2:1. The gel was run for 15 min.

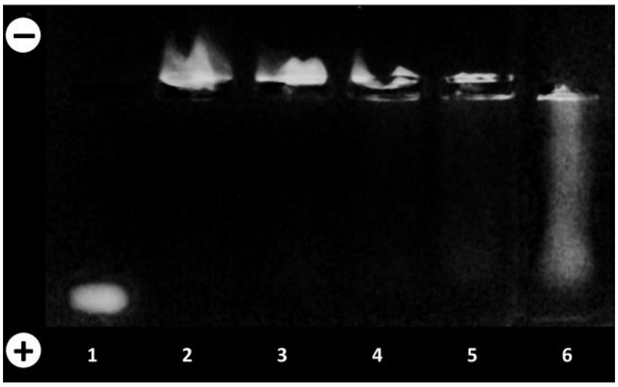

Figure S4. Gel electrophoresis results for DNA oligonucleotide complex with P11 polymer. Lane 1: free oligonucleotide; 2–6: complexes with P11 of various concentrations: 2, 1, 0.7, 0.3, and 0.2 g·L^{-1}, correspondingly. The polymer:oligonucleotide ratio (volume to volume) was 2:1. The gel was run for 15 min.

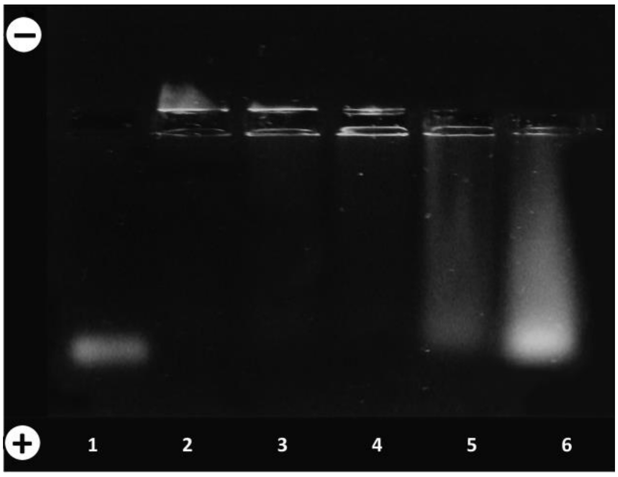

Figure S5. Gel electrophoresis results for DNA oligonucleotide complex with P12 polymer. Lane 1: free oligonucleotide; 2–6: complexes with P12 of various concentrations: 2, 1, 0.7, 0.3, and 0.2 g·L^{-1}, correspondingly. The polymer:oligonucleotide ratio (volume to volume) was 2:1. The gel was run for 20 min.

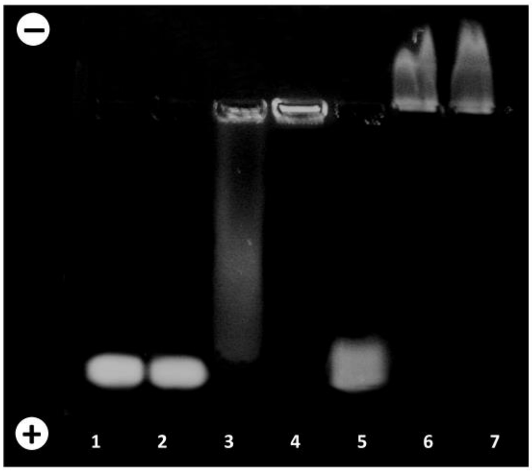

Figure S6. Gel electrophoresis results for DNA oligonucleotide complex with new polymers and composites. Lane 1: free oligonucleotide; 2–4: P01 based composites; 5: P02 based composite; 6 and 7: P01 and P02 polymers without silica, correspondingly. The concentration of the polymers was 0.4 $g \cdot L^{-1}$, silicic acid: 12.5, 10, 7.5, and 10 mM for lanes 2–5 lanes, correspondingly, and oligonucleotide: 10 μM. The polymer (composite):oligonucleotide ratio (volume to volume) was 2:1. The gel was run for 28 min.

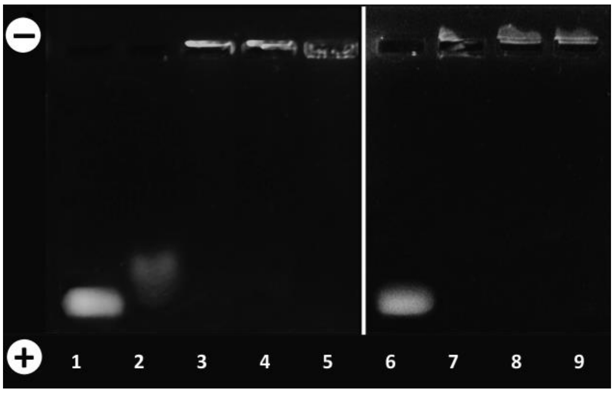

Figure S7. Gel electrophoresis results for DNA oligonucleotide complex with new polymers and composites. Lanes 1, 6: free oligonucleotide; 2, 3: P11 based composites; 4: P12-based composite; 5: P22-based composite; 7, 8, and 9: P11, P12, and P22 polymers, correspondingly. Concentration of the polymers was 0.4 $g \cdot L^{-1}$, silicic acid: 10, 7.5, 7,5, and 10 for lanes 2–5, correspondingly, and oligonucleotide: 10 μM. The polymer (composite):oligonucleotide ratio (volume to volume) was 4:1. The gel was run for 28 min
